# Prevalence of HIV, HBV and HCV among livestock merchants and slaughterhouse workers in Ibadan, Nigeria

**DOI:** 10.4314/ahs.v24i1.4

**Published:** 2024-03

**Authors:** Adewale V Opayele, Olamide T Arege, Adedayo O Faneye, David O Olaleye, Georgina N Odaibo

**Affiliations:** 1 Department of Virology, College of Medicine, University of Ibadan, Ibadan, Nigeria; 2 Department of Molecular Genetics and Genomics, Wake Forest School of Medicine, North Carolina, USA

**Keywords:** Human immunodeficiency virus, viral hepatitis, slaughterhouse workers

## Abstract

**Background:**

Most studies on viral infections among livestock handlers have focused on occupational exposure from inadvertent contact with infected animals. Consequently, little emphasis is given to the effect of their lifestyle on the acquisition of other blood-borne viruses.

**Objectives:**

To determine the prevalence and assess risk factors for HIV, HBV and HCV infections among livestock handlers in Ibadan, Nigeria.

**Methods:**

Blood samples were collected from 265 livestock handlers between October 2016 to April 2017 in Ibadan. The samples were tested for the presence of antibodies to HIV and HCV; and surface antigen to HBV using ELISA. Structured questionnaire was administered to collect information on risk factors associated with the transmission of these viruses. Data analysis was carried out using Chi-square test and logistic regression to determine the association between risk factors and predictors of infection (p < 0.05).

**Results:**

Of 265 participants, 11 (4.2%), 29 (10.9%) and 13 (4.9%) individuals tested positive for HIV, HBV and HCV infections respectively. Two (0.8%) of the participants were coinfected with HIV and HBV while 1(0.4%) was coinfected with both HBV and HCV. Individuals who travelled frequently in the course of Livestock trades had a higher rate of HIV infection.

**Conclusions:**

A high Infection with HIV, HBV and HCV is common among the study participants. There is a need for continued surveillance and awareness creation on preventive measures against these viruses.

## Introduction

Human immunodeficiency virus (HIV), a member of the retrovirus family is one of the leading causes of morbidity and mortality in humans. Worldwide, about 38.4 million people are living with the virus and over 2 million people have died of the infection^1^. Sub Saharan Africa bears one of the highest burdens of the epidemic. In 2017, 75% of deaths and 65% of new infections occurred on the continent where about 71% of people living with HIV (PLWH) reside^2^. In Nigeria, there are about 1.9 million people living with the virus 3. Hepatitis B virus (HBV), a member of the Hepadnaviridae family is a partially double-stranded DNA virus with a small genome (3.2 kb). Since its discovery in 1965, HBV infection has remained a threat to public health 4. Chronic HBV infection results in serious liver disease including liver cirrhosis and hepatocellular cancer (HCC) if untreated 5. Hepatitis C virus (HCV) on the other hand is a positive sense, single-stranded RNA virus belonging to the Flaviviridae family. Like HBV, Chronic HCV infection may progress to severe liver diseases including cirrhosis and hepatocellular carcinoma 6. Globally, about 354 million people are estimated to have hepatitis B or C which are the most common cause of viral hepatitis-related deaths.^7^

The risk factors and mode of transmission of HIV, HBV and HCV are similar. Their shared routes of transmission include through sexual contact, parenteral, mother-to-infant and other horizontal routes that allow access to blood and other body fluids 8. It is therefore common to encounter coinfection with the three viruses in populations at risk of exposure 9. Over the years, there has been a progressive decrease in mortality from AIDS-related opportunistic infections and malignancies due to the success of antiretroviral therapy (ART). However, this success has uncovered new set of challenges due to the emergence of chronic liver disease as the leading cause of morbidity and mortality among HIV/HBV/HCV coinfected population 10. HIV coinfection with HBV/HCV poses a serious challenge to (ART) as affected individuals do not respond well to treatment like HIV monoinfected individuals. This population is also at risk of potential drug interactions due to complexities involved in treatment 11.

Individuals whose jobs involve accidental exposure to percutaneous injury from cuts by sharp objects are among high-risk groups for these infections. Butchers, livestock handlers and other slaughter house workers fall under this category 12,13. Often times, most studies on the transmission of viral infections among this population focus on zoonotic diseases transmitted through trading and processing of infected animals. This focus downplays the public health importance of other transmissible viral infections among these populations. In Nigeria, many livestock markets and slaughter houses violate standard hygiene and disinfection practices 14 and majority of the workers are uneducated, thus, practices that allow contact with body fluids from contaminated sharp objects such as sharing of cutlasses, knives and razors, frequent hand cuts, improper wound dressing and other forms of indifference to hazardous biological fluids that may constitute risks for infectious disease transmission are common 12.

The prevalence of HIV, HBV and HCV in mono, dual or triple infection has been reported in several populations in Nigeria including Sickle Cell Disease Patients 15, pregnant women 16, blood donors 17, healthcare workers 18, sex workers 19 and men who have sex with men including transgenders 20. However, there is paucity of information on coinfection of these viruses among livestock merchants and slaughterhouse workers. This study was therefore carried out to determine the burden of HIV, HBV and HCV infections among this high-risk population in Nigeria.

## Materials and Methods

### Study area and population

This cross-sectional study was carried out in Ibadan, a city in Oyo State, southwest, Nigeria with over 3.7 million inhabitants. Ibadan is the largest geographical municipal area in Nigeria 21 and the third most populous city in the country. This study was carried out among workers in Akinyele Cattle Market (AKCM), Bodija Abattoir (BA) and the University of Ibadan (UI) livestock farm. Most ruminants slaughtered for meat in the southern region of Nigeria are reared in the northern states of the country and some are imported from other neighbouring countries like Niger and Chad. AKCM is the main market in southern Nigeria where cattle from the northern states of the country are received and traded. Similarly, Bodija abattoir is the largest slaughter facility in Ibadan city and the whole of south-western Nigeria. Hundreds of livestock are slaughtered and processed daily in this abattoir 22. Due to the various activities in the slaughter facilities, a large number of livestock merchants, butchers, meat retailers and their respective apprentices carry out various meat processing and handling in the slaughter facility. Workers at the UI livestock farm also care for and slaughter livestock for use in research projects and consumption within the University community.

### Sample and data collection

A total of 254 blood samples were collected from October 2016 to April 2017 at the 3 study sites. Consenting participants were enrolled into the study and interviewed using structured questionnaire to obtain information on socio-demographic profiles, occupation, travel history, vaccination status, knowledge of blood borne and sexually transmitted viral diseases including HIV, HBV and HCV.

Five milliliters of venous blood samples were collected from each study participant into a pre-labelled EDTA vacutainer tubes and transported in a cold box to the Department of Virology, University College Hospital, Ibadan where blood samples were centrifuged at 3000 rpm for 15 mins, plasma separated and stored in aliquots at −20°C until analyzed. The study protocol was reviewed and approved by the UI/UCH ethics committee (Number UI/EC114/0274).

### Laboratory analysis

All tests were conducted in the serology laboratory of the Department of Virology, an ISO 15189 (SANAS) accredited facility. Stored plasma samples were tested for the presence of antibodies to HIV and HCV as well as surface antigen to HBV using commercially available 3rd and 4th generation ELISA kits.

For the diagnosis of HIV Infection, plasma samples were tested using a fourth generation ELISA (GenScreen Ultra HIV Ag-Ab, Bio-Rad, Paris) with high sensitivity to HIV antigen and antibodies. Initially reactive samples were re-tested using same assay to confirm positivity. The diagnosis of HBV Infection was carried out using a surface antigen detection ELISA (Monolisa, Bio-Rad, Paris) and positive samples re-tested using the same kit. Antibodies to HCV were detected using a third generation ELISA (HCV-Ab, Dia.PRO, Italy). Positive samples were re-tested using the same kit. All assays were carried out according to manufacturer's instruction.

### Data analysis

Data were cleaned and analyzed using SPSS version 22. Inferential and descriptive statistics were used for results presentation and interpretation. Logistic regression was carried out to assess the relationship between risk factors as predictors of viral infections among study population. Associations between risk factors and viral infections was also determined using Chi-Square test at significance level below 0.05.

## Results

The socio-demographic characteristics of respondents are shown in Table 1. In total, 265 participants were enrolled in this study. Majority of the population according to gender, ethnicity, marital status and occupation were males (92.8%), Yoruba (68.7%), married (97.0%) and Livestock trader (74.7%) respectively. More than half of the study participants were from the southern part of the country (68.3%). Most (83.4%) had prior knowledge of HIV and its modes of transmission while only a few (15.1%) knew about HBV and HCV transmission and only 1.1% reported to have been vaccinated against HBV.

**Table 1:** Sociodemographic characteristics of study participants (N-265)

Characteristic	Frequency (%)
Gender	
Male	246 (92.8)
Female	19 (7.2)
Ethnicity	
Hausa	79 (29.9)
Igbo	2 (0.8)
Ijaw	2 (0.8)
Yoruba	182 (68.7)
Marital Status	
Married	257 (97.0)
Unmarried	5 (1.9)
Divorced	3 (1.1)
Occupation	
Butcher	56 (21.1)
Livestock trader	198 (74.7)
Others	4.2 (4.2)
Place of Birth	
Northern States	84 (31.7)
Southern States	181 (68.3)
Aware of HIV transmission	
Yes	221 (83.4)
No	44 (16.6)
Aware of HBV and HCV Transmission	
Yes	40 (15.1)
No	225 (84.9)
Vaccinated against HBV	
Yes	3 (1.1)
No	261 (98.5)
Non-respondent	1(0.4)

Of the 265 study participants, 11 (4.2%), 29 (10.9%) and 13 (4.9%) tested positive for HIV, HBV and HCV infections respectively.

**Figure 1:**
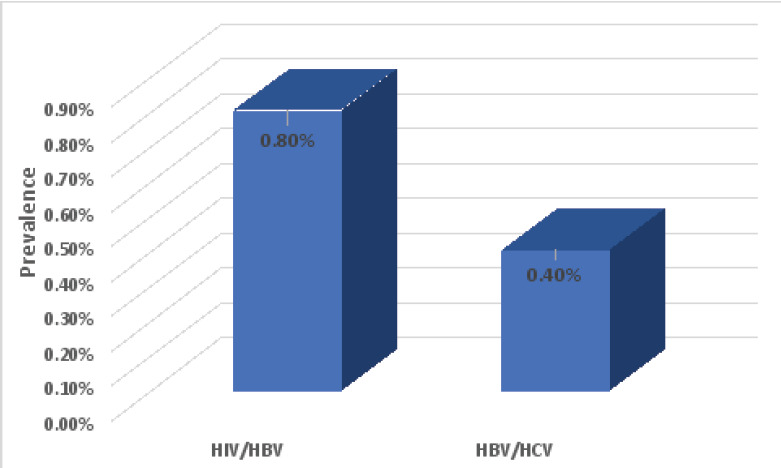
Prevalence of HIV, HBV and HCV coinfections among study participants

Overall, the rate of coinfection in this study was 1.2% (3/254) among study participants. Two (0.8%) were positive to both HIV and HBV infections, while one (0.4%) was positive for both HBV and HCV (Figure 1). No coinfection with HIV and HCV or HI/HBV/HCV triple infection was found in this study.

Table 2 shows the prevalence of HIV, HBV and HCV infection by age group and occupation of the participants. Although, there was no significant difference in prevalence among the various age groups, the 41-50 age group had a highest prevalence rate for the three infections. Similarly, livestock traders had a highest rate of infection for the three viruses. A statistically significant difference was found in the rate of HIV infection among the various occupations.

**Table 2:** Prevalence of HIV, HBV and HCV by age group and occupation among study participants

		HIV Infected		HBV Infected		HCV Infected	
	No	No (%)	p-value	No	p-value	Positive	p-value
	Tested	positive		(%) Positive		(%)	
**Age (yrs)**							
20-30	14	0 (0.0)	0.325	3 (1.1)	0.178	0 (0.0)	0.93
31-40	55	0 (0.0)		6 (2.3)		3 (1.1)	
41-50	77	5 (1.9)		13 (4.9)		4 (1.5)	
51-60	52	3 (1.1)		3 (1.1)		2 (0.8)	
61-70	43	1 (0.4)		3 (1.1)		3 (1.1)	
>70	24	2 (0.8)		1 (0.4)		1 (0.4)	
Total (%)	265	11 (4.2)		29 (10.9)		13 (4.9)	
**Occupation**							
Butchers	56	0 (0.0)	0.019	8 (3.0)	0.477	1 (0.4)	0.205
Livestock Traders	198	9 (3.4)		19 (7.1)		12 (4.5)	
Others	11	2 (0.8)		2 (0.8)		0 (0.0)	
**Total (%)**	265	11 (4.2)		29 (10.9)		13 (4.9)	

Significant association was determined via two-sided Chi-square test (p < 0.05).

**Figure 2:**
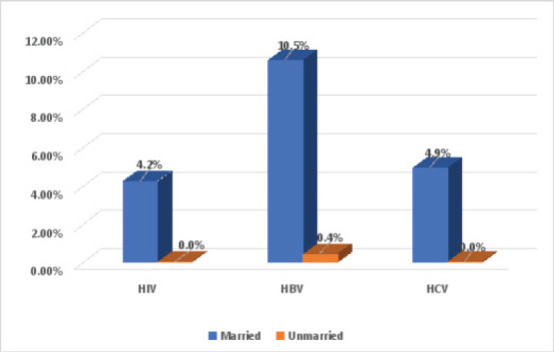
Percentage Distribution of HIV, HBV and HCV infections according to marital status among study participants at p≤0.05. p=0.709 for HIV, p=0.610 for HBV and p=0.665 for HCV

HBV infection was highest among both married and unmarried study participants. There was no significant difference in the rates of infection by marital status. Figure 2. Significant association was determined via two-sided Chi-square test (p < 0.05).

Risk factor column indicates potential variables associated with HBV infection among study population. Odds Ratio and 95% CI determined via logistic regression measures the odds of association between HBV infection and risk factors among study population. P-value determined via logistic regression. (P < 0.05) **; (P < 0.1) *. No significant relationship was found between risk factors and infections with HIV or HCV among study population.

## Discussion

The prevalence of HIV among livestock merchants and slaughter house workers in this study was 4.3%, this is higher than 1.4% reported among the general population in Nigeria 3. It is also much higher than the prevalence obtained in Oyo state (0.9%) during the 2019 national survey 3. Our literature search shows that there is a paucity of information on the prevalence of HIV among slaughter house workers in Nigeria. Consequently, the rate of HIV infection obtained from this population was compared to those obtained from hospital workers, another high-risk group. The rate obtained in this study is lower than 9.7% obtained among healthcare workers in Ibadan 18. This further shows the need to pay attention to high-risk populations when planning for HIV control programs.

The Prevalence of hepatitis B infection in this study was 10.9%, this is similar to 12.2% reported in a recent National Survey in Nigeria 23. However, our result is slightly higher than 9.4% previously reported among butchers in Ibadan 12. Our findings is lower than those obtained from some other populations in Ibadan including 16.3% among pregnant women 24, 18.3% among hairdressers 25 and 13.1% among blood donors 26. The difference may imply gains in intervention efforts geared towards reducing the burden of HIV, a disease with similar route of infections with HBV, some of which include increased awareness of transmission and prevention measures 27.

At the moment, there is no country level population-based data on the prevalence of HCV. However, studies have been carried out among various populations in different states in the country, all with varying prevalence. The HCV infection rate of 4.9% obtained in this study is higher than those reported among some low-risk population groups in the country, including 1.7% found among pregnant women in Ibadan 28, 1% among children aged 10-18 years in Enugu 29 and 0.40% among healthy university undergraduates in Ogbomosho, Nigeria 30. The rate is however lower than what was reported among some other groups at higher risk of infection, including 37.9% among patients presenting with STDs in Ibadan 31.

A low HBV/HCV coinfection rate of 0.4% was found in this study. This agrees with those from other studies in Ibadan where similarly low rate of 0.1% among pregnant women and 1% among PLWH were reported 32,33. Opaleye et al also reported a low HBV/HCV co-infection rate of 1.1% among blood donors from other south western part of Nigeria 34. Similarly, a low rate of HIV/HBV coinfection of 0.8% was found in this study. This is very close to 0.85% reported among pregnant women in Oyo state 16 and lower than the 8.9% and 11.9% reported among pregnant women and PLWH respectively previously at the University College Hospital, Ibadan between 2004 and 2007 when the prevalence of HIV was over 4% in the country 32,33,35. Recently, a higher rate of HIV/HBV coinfection (16.1%) was reported by Opaleye et al. in a study among HIV positive individuals in various hospitals across different states in the southwestern region of Nigeria. 36. Other studies carried out among other populations in the country have reported similar varying rates, most still higher than what we found in this study. For instance, Omatola et al. reported 3.5% among PLWH in Anyigba, Kogi State, North-Central Nigeria 37 while Magaji et al. reported a relatively higher HIV/HBV coinfection rate of 12.6% among pregnant women in Jos, Nigeria 38. The difference in the rates reported in various states may be due to the difference in prevalence of these viruses in the states.

Majority of study participants had prior knowledge of HIV and its modes of transmission while only a few had knowledge of HBV and HCV which are viruses that share the same routes of transmission. This may be due to the lopsided attention paid to HIV intervention strategies over the years 39. Knowledge of HIV transmission had a protective effect against HBV acquisition among study participants based on its significant p-value and reported 95% CI determined via logistic regression. Age had slight effect onf HBV transmission and this could be a consequence of sexually active population recruited for this study.

The inclusion of HBV and HCV in this intervention may serve to reinforce advocated precautionary measures in place to protect members of the public against these viruses. Also, only 1.1% of the study participants reported to have been vaccinated against HBV, indicating a need to strengthen awareness about the availability of HBV vaccine 40. Participants over 40 years of age had the higher rates of infections for the three viruses. This could be because they are likely to have had a higher exposure to predisposing factors and sexual intercourse, especially those in a polygamous relationship. Also, livestock traders had similar rates of infection with the three viruses. This may imply an increase in risk of exposure due to frequent travels and nomadic lifestyle of some of these traders. This is necessitated by travels outside their permanent residencies for purchase of livestock and the search of edible pasture for their herd. Sexual behaviours mainly from having extramarital affairs at different city stops may have also contributed to the rates of HIV, HBV and HCV infections among this study population 41. The rate of HBV infection was highest among married participants while HCV infection was highest among unmarried participants.

This study is limited by the low numbers of participants recruited in each of the two major occupational groups. In future studies, including a larger number of participants will be beneficial. Butchers had higher exposures that predisposes them to cuts and contact with infected blood while livestock traders who slaughtered and processed meat less frequency were involved in more travels and time away from their families, a risk for being involved with multiple sexual partners. Although, the rate of infection among livestock traders is higher than that of butchers in this study, conclusions on the rates of infection among the two occupational groups need to be interpreted with caution since we did not recruit equal number of participants from these occupations. Furthermore, the occupational roles of study participants are male dominated; therefore, a very high proportion of males were sampled in comparison to underrepresented females. Therefore, gender related conclusions cannot be made from our results.

This study appears to be first serological investigations of triple infections with HIV, HBV and HCV among this important population in Nigeria. Although, we found low rates of HIV/HBV and HBV/HCV coinfections among study participants, monoinfection with HIV, HBV and HCV was still common among livestock traders and slaughter house workers in Ibadan. This emphasises the need for continued surveillance. Additionally, there is a need to strengthen awareness of preventive measures against these viruses, including the availability of a preventive HBV vaccine.

## Figures and Tables

**Table 3:** Relationship between risk factors (predictors) and HBV infection

Risk factor	Odds Ratio (95% CI)	p-value
Age	0.97 (0.93 - 1.00)	0.0764*
Occupation	0.89 (0.34 - 2.31)	0.8185
Knowledge of HBV transmission	0.19 (0.02 - 1.47)	0.1116
Knowledge of HIV transmission	0.38 (0.15 - 0.96)	0.0397**
